# Persistence of two coronaviruses and efficacy of steam vapor disinfection on two types of carpet

**DOI:** 10.1186/s12985-024-02478-9

**Published:** 2024-09-02

**Authors:** Jinge Huang, Angela Fraser, Xiuping Jiang

**Affiliations:** Department of Food, Nutrition, and Packaging Sciences, Clemson University, 228A Life Science Facility, Clemson, SC 29634 USA

**Keywords:** Bovine coronavirus, Human coronavirus OC43, Carpet, Persistence, Steam vapor disinfection

## Abstract

**Background:**

Coronaviruses, a group of highly transmissible and potentially pathogenic viruses, can be transmitted indirectly to humans via fomites. To date, no study has investigated their persistence on carpet fibers. Establishing persistence is essential before testing the efficacy of a disinfectant.

**Methods:**

The persistence of BCoV and HCoV OC43 on polyethylene terephthalate (PET) and nylon carpet was first determined using infectivity and RT-qPCR assays. Then, the disinfectant efficacy of steam vapor was evaluated against both coronaviruses on nylon carpet.

**Results:**

Immediately after inoculation of carpet coupons, 32.50% of BCoV and 3.87% of HCoV OC43 were recovered from PET carpet, compared to 34.86% of BCoV and 24.37% of HCoV OC43 recovered from nylon carpet. After incubation at room temperature for 1 h, BCoV and HCoV OC43 showed a 3.6 and > 2.8 log_10_ TCID_50_ reduction on PET carpet, and a 0.6 and 1.8 log_10_ TCID_50_ reduction on nylon carpet. Based on first-order decay kinetics, the whole gRNA of BCoV and HCoV OC43 were stable with *k* values of 1.19 and 0.67 h^− 1^ on PET carpet and 0.86 and 0.27 h^− 1^ on nylon carpet, respectively. A 15-s steam vapor treatment achieved a > 3.0 log_10_ TCID_50_ reduction of BCoV and > 3.2 log_10_ TCID_50_ reduction of HCoV OC43 on nylon carpet.

**Conclusion:**

BCoV was more resistant to desiccation on both carpet types than HCoV OC43. Both viruses lost infectivity quicker on PET carpet than on nylon carpet. Steam vapor inactivated both coronaviruses on nylon carpet within 15 s.

## Introduction

Coronaviruses, a group of RNA viruses, can infect various mammalian species, including humans. Among them, betacoronaviruses play a significant role in causing infection. Human betacoronaviruses, such as human coronavirus (HCoV) OC43, Middle East Respiratory Syndrome coronavirus (MERS-CoV), severe acute respiratory syndrome coronavirus (SARS-CoV), and SARS-CoV-2, can cause infections ranging from asymptomatic to severe [1–3]. In cattle, seroprevalence studies indicate that over 90% of cattle are exposed to bovine coronavirus (BCoV) during their lifetime, causing both respiratory and enteric infections [4]. Importantly, due to their close antigenic and genetic relatedness, betacoronaviruses including BCoV, HCoV OC43, SARS-CoV and SARS-CoV-2 exhibit the capacity for interspecies transmission [4–6]. For example, the original host of SARS-CoV was possibly identified as bats [7]. Betacoronaviruses cause a more infection so are a good surrogate for SARS-CoV-2, which can cause a severe infection [7, 8].

Coronaviruses are primarily transmitted through direct contact with aerosols and droplets, with indirect transmission possible when persistent in the environment [9]. Multiple laboratory studies confirmed SARS-CoV-2 persistence on fomites (e.g., furniture, remote controls, countertops) but persistence varies widely depending on surface material [9–12]. For example, stainless steel coupons, a non-porous material, inoculated with SARS-CoV-2 showed a 1 log_10_ TCID_50_ reduction in 1 to 2 days, whereas cotton, a porous material, showed more wide-ranging results, from < 1 log_10_ to > 4 log_10_ TCID_50_ reduction in 1 day [10, 13]. These variations suggest the need to determine persistence of coronaviruses on a wider range of surface materials.

Hard flooring has been reported to be a reservoir for viral particles [14]. These particles can be re-suspended in the air through mechanical agitation (e.g., walking and vacuuming) [14, 15]. Less is known about porous flooring materials, such as carpet, which is widely used in public spaces, as it provides comfort, insulates sound, and prevents falls so is often impractical to replace with non-porous materials, such as hard flooring [16, 17]. Carpet is unique due to its composition and structure, which presents special challenges when assessing recovery, persistence, and disinfection of viruses. Traditionally, carpet is cleaned by frequent vacuuming [18, 19]. However, the process of vacuuming might unintentionally resuspend viral particles, dispersing them into the surrounding environment [15, 20]. To disinfect carpet after a bodily fluid event, the U.S. Centers for Disease Control and Prevention (CDC) recommends steam cleaning [20, 21]. Steam vapor is reportedly effective against feline calicivirus (FCV) with a > 3 log_10_ plaque-forming-unit reduction on wool and nylon carpet within 1.5 min, and bacteriophage Phi6 on polyethylene terephthalate carpet within 1 min [15, 22]. While steam vapor has demonstrated efficacy against some viruses on carpet, its efficacy against coronaviruses, particularly betacoronaviruses, has yet to be confirmed.

The detection of viruses from porous materials is challenging, hence, investigators often rely on the degradation of viral RNA as the sole metric for assessing concentrations of virus in the environment [23, 24]. While this approach provides valuable insights into persistence of the viral genome and structural integrity, it does not provide an assessment of coronavirus infectivity. Furthermore, coronaviruses have exhibited sensitivity to recovery methodology, e.g., detergents used for recovery [25], supporting the need to identify a better recovery method.

We aimed to fill these knowledge gaps by first evaluating the persistence of two pathogenic betacoronaviruses, BCoV and HCoV OC43, on two types of carpet -- polyethylene terephthalate (PET) and nylon. Then we tested the disinfection efficacy of steam vapor against these two coronaviruses on nylon carpet. Our findings can be used to inform disinfection strategies on porous materials, such as carpet.

## Materials and methods

### Virus propagation and assays

The cell line and virus were cultured as previously described [26]. Briefly, human rectal tumor (HRT-18G) cells, CRL-11663 were acquired from American Type Culture Collection (ATCC)] and cultured in Dulbecco’s Modified Eagle Medium (DMEM) containing 4.5 g/L glucose, 3% low-endotoxin heat-inactivated fetal bovine serum (FBS), 100 U/L penicillin, and 100 mg/L streptomycin at 37 °C and 5% CO_2_. Ninety percent (90%) confluent monolayers of HRT-18G cells were infected with bovine coronavirus (BCoV) strain Mebus (acquired from BEI Resources, NR-445), or HCoV OC43 (acquired from ATCC, VR-1558) at a multiplicity of infection (MOI) of 0.01, then incubated at 37 °C for five days. BCoV and HCoV OC43 were then harvested from cell lysates by three freeze-thaw cycles followed by centrifugation for 10 min at 5,000 × g and 4 °C. BCoV and HCoV OC43 stocks at ca. 10^8^ 50% tissue culture infectious dose (TCID_50_)/mL were aliquoted and stored at -80 °C. HRT-18G cells were passaged less than 30 times for all experiments.

Infectious BCoV and HCoV OC43 were quantified by TCID_50_ assay as previously described, with modifications [27]. Briefly, monolayers of HRT-18G cells at 90% confluency were infected with 100 µL of viral samples at 33 °C for 1 h in a humidified 5% CO_2_ incubator. This was followed by the addition of 100 µL of DMEM containing 4.5 g/L glucose, 2% low-endotoxin FBS, 100 U/L penicillin, and 100 mg/L streptomycin. After incubating at 33 °C in a humidified 5% CO_2_ incubator for seven days, the virus titer was determined by the improved Kärber method [28]. To test cell line susceptibility to infection and viability, BCoV or HCoV OC43 stock and phosphate buffer saline (PBS) were used as positive and negative controls, respectively.

### Carpet coupon preparation and selection of steam cleaner

Two common and popular commercial carpet materials, which accounted for > 50% of production in the United States, PET carpet (Profusion 20^®^, Shaw Inc., GA, USA) and nylon carpet (Color Accent^®^, Shaw Inc., GA, USA), were tested. The choice of these two carpets was made according to the Carpet and Rug Institute guidelines - CRI Test Method 114 (carpet-rug.org) and expert opinion. Both PET and nylon carpet had no antimicrobial coating and were low pile with fiber pile thickness at 3.58 and 2.92 mm, respectively. Carpet samples were cut into 5 × 5 cm^2^ coupons with a mechanical cutting die (model 1500, Freeman Schwabe, OH, USA) (kindly provided by Dr. Daniel Price, Interface Inc., GA, USA) then dusted by gloved hand to remove loose fibers. To remove additional residue, coupons were scoured using a boiling solution of 5 g/L Tergitol N-101 (Spectrum Chemical Inc., New Brunswick, NJ) and 5 g/L of Na_2_CO_3_ (Fisher Scientific, MA, USA), then rinsed with cold tap water until visibly clean. Before testing, carpet coupons were autoclaved on a 20-min dry cycle and cooled at room temperature overnight.

A household steam cleaner (IVASTEAMR20, Ivation, NJ, USA) that can generate 170 °C, 29–65 psi steam in its boiler was used with a small round head (4 cm in diameter) to test the efficacy of steam vapor. To prevent cross-contamination during steam vapor treatment, the head of a steam cleaner was wrapped with a sterile terry cloth folded into four layers.

### Persistence of coronaviruses on carpet

BCoV and HCoV OC43 were prepared at ca. 10^8^ TCID_50_/mL with 5% heat-inactivated FBS representing soil load. Each pre-cut carpet coupon was inoculated with 100 µL suspension of either BCoV or HCoV OC43 then kept for 120 min under a biosafety cabinet (Model 1300 A2, Thermo Fisher, MD, USA) at room temperature with a relative humidity at 30–50%. After kept for 0, 10, 20, 30, 60, 90 and 120 min, three coupons were immediately transferred into a flask with 100 mL of PBS plus 0.02% Tween-80 [22, 25]. All flasks were ultrasonicated for 1 min at 40 kHz (Model FS110, Fisher, PA, USA) then vigorously shaken by hand for 30 s. Samples were recovered and concentrations of each coronavirus in each sample were assayed as described above. The percent recovery rate was calculated from titer values without logarithm transformation, while other data was logarithm transformed for analysis. The detection limit was 2.6 log_10_ TCID_50_/coupon.

### RT-qPCR and RNase-treated RT-qPCR

Viral genome RNA (gRNA) extraction was performed as previously described [22]. Briefly, BCoV and HCoV OC43 gRNAs were extracted from 0.15 mL of carpet samples using an ENZA viral RNA kit (Omega Bio-Tek, GA, USA) per manufacturer instructions. After extraction, gRNAs were stored at -80 °C before further analysis.

Reverse transcription-quantitative polymerase chain reaction (RT-qPCR) was performed for BCoV and HCoV OC43 separately to determine the loss of viral gRNAs using a Platinum SYBR Green PCR kit (Invitrogen, CA, USA). For RT-qPCR analysis, the forward and reverse primer sequences were CTGGAAGTTGGTGGAGTT and ATTATCGGCCTAACATACATC for BCoV, respectively, and CGATGAGGCTATTCCGACTAGGT and CCTTCCTGAGCCTTCAATATAGTAACC for HCoV OC43, respectively. The standard curves were individually prepared for BCoV and HCoV OC43 by 7-step, 10-fold dilutions of virus stocks.

To assess the structural integrity of the viral capsid, the exposed gRNA, due to capsid cleavage, was removed by RNase I pretreatment of samples prior to RNA extraction [29]. Briefly, 0.1 U/µL RNase I (Thermo Fisher, MD, USA) was mixed with 250 µL of samples and incubated at 37 °C for 15 min. RNA extraction was performed immediately after the RNase-I pretreatment as described above.

### Determination of disinfection efficacy of steam vapor against coronaviruses on nylon carpet

As both coronaviruses did not persist on the PET carpet, disinfection efficacy of steam vapor was determined only on nylon carpet following the protocol in a previous study with minor modifications [22]. Briefly, each pre-cut coupon was inoculated with a 100 µL suspension of either BCoV or HCoV OC43 then dried for 1 h at room temperature at a relative humidity at 30–50%. To evaluate steam vapor efficacy, the steam cleaner was preheated, then the wrapped head and hose were saturated with steam for 10 s per manufacturer instructions. The cloth wrapped head was changed between samples to avoid cross-contamination. Coupons were scrubbed vertically for 15 s with steam. All coupons were transferred into a flask with 100 ml of PBS plus 0.02% Tween-80 to elute virus from carpet coupons. To evaluate the effect of scrubbing on virus inoculum, three coupons were scrubbed using the wrapped head of steam cleaner without heat as the scrubbed controls, while three unscrubbed coupons were immediately transferred to elution buffer after drying to evaluate desiccation effect. All flasks were ultrasonicated for 1 min at 40 kHz then vigorously shaken by hand for 30 s to recover virus inoculum from carpet coupons. Titers of BCoV and HCoV OC43 in samples were assayed by TCID_50_ assay as described above. A 3 log_10_ TCID_50_/coupon reduction was used as the benchmark for successful disinfection efficacy, which is in accordance with guidelines from the U.S. Environment Protection Agency [30]. The temperature of steam vapor was measured using type T thermocouples (HotMux, DCC Corporation, NJ, USA).

### Carpet absorption capacity

To measure the hydrophobicity of carpet fibers, the water absorption capacity of carpet fibers was tested as described previously [31]. Briefly, the carpet fibers were cut from the coupons using a disposable scalpel (Sklar, PA, USA) then 0.1 g of fibers were packed in a 2-mL microcentrifuge tube (Fisher Scientific, CA, USA). PET and nylon fibers were thoroughly mixed with an indicator dye, safranin solution (0.1%), in increments of 0.05 mL until the carpet was saturated and removed afterwards. The weight of residual liquid was obtained by subtracting the weight of the empty microcentrifuge tube from the weight of the microcentrifuge tube after treatment.

### Statistical analysis

Six replicates were tested in two independent tests to determine persistence of each coronavirus, whereas 2 independent tests with 5 replicates (*N* = 10) were conducted to test disinfection efficacy of steam vapor against each of the two coronaviruses. Microbial reductions were calculated using log_10_ (*N*_*0*_*/N*_*d*_), where *N*_0_ is the average coronavirus titers from the samples at 0 min after drying or the control samples, and *N*_*d*_ is the average coronavirus titers from the samples at different sampling times or the steam-treated samples.

For RNA determination, the first-order decay rate constants (*k*) were calculated using the following Eq. (1), where *N*_0_ is the average amount of coronavirus RNA at 0 h and *N*_*t*_ the average amount of coronavirus RNA at time (t). *k* values were calculated by plotting ln (*N*_*t*_*/N*_*0*_) versus time (t) and calculating the slope, its standard error, with β_0_ as the intercept.

1 $$\:\text{ln}\left(\frac{{N}_{t}}{{N}_{0}}\right)={\beta\:}_{0}-kt$$

Statistical analysis was performed using a one-way multiple-comparison ANOVA and Tukey’s test to determine the relationship between steam vapor and virus titer reduction. All results were expressed as mean ± standard deviation. Statistical significance was defined as a *p*-value of < 0.05. Statistical analyses were conducted using GraphPad Prism 6.01 (GraphPad Software, Inc., CA, USA).

## Results

### Persistence of infectious coronaviruses on carpet

The immediate recovery rate of BCoV from PET and nylon carpet, with an initial inoculum level at 4.2 × 10^6^ – 1.8 × 10^7^ TCID_50_/coupon, was 32.50% and 34.86%, respectively. This was significantly higher than the recovery rate of HCoV OC43 from PET and nylon carpet, with initial inoculum level at 7.1 × 10^6^ – 2.1 × 10^7^ TCID_50_/coupon, which was 3.87% and 24.37%, respectively (Table 1).

**Table 1:** Recovery rate of BCoV and HCoV OC43 from carpets

Virus	Recovery rate (%) ^a^			
	PET carpet		Nylon carpet	
	Viability	gRNA copies	Viability	gRNA copies
BCoV	32.50 ± 14.32^A^	52.32 ± 35.43^A^	34.86 ± 12.44^A^	57.64 ± 6.54^A^
HCoV OC43	3.87 ± 2.02^B^	40.84 ± 7.57^A^	24.37 ± 6.21^B^	28.54 ± 8.71^B^

^a^ The percent recovery rate was calculated from titer values without logarithm transformation. Data are expressed as mean ± standard deviation (SD) from triplicates from each of 2 independent experiments. Values with different letters for the same fiber (e.g., A, B) indicate significant difference (*p* < 0.05) in Tukey’s test grouping

**Fig. 1:**
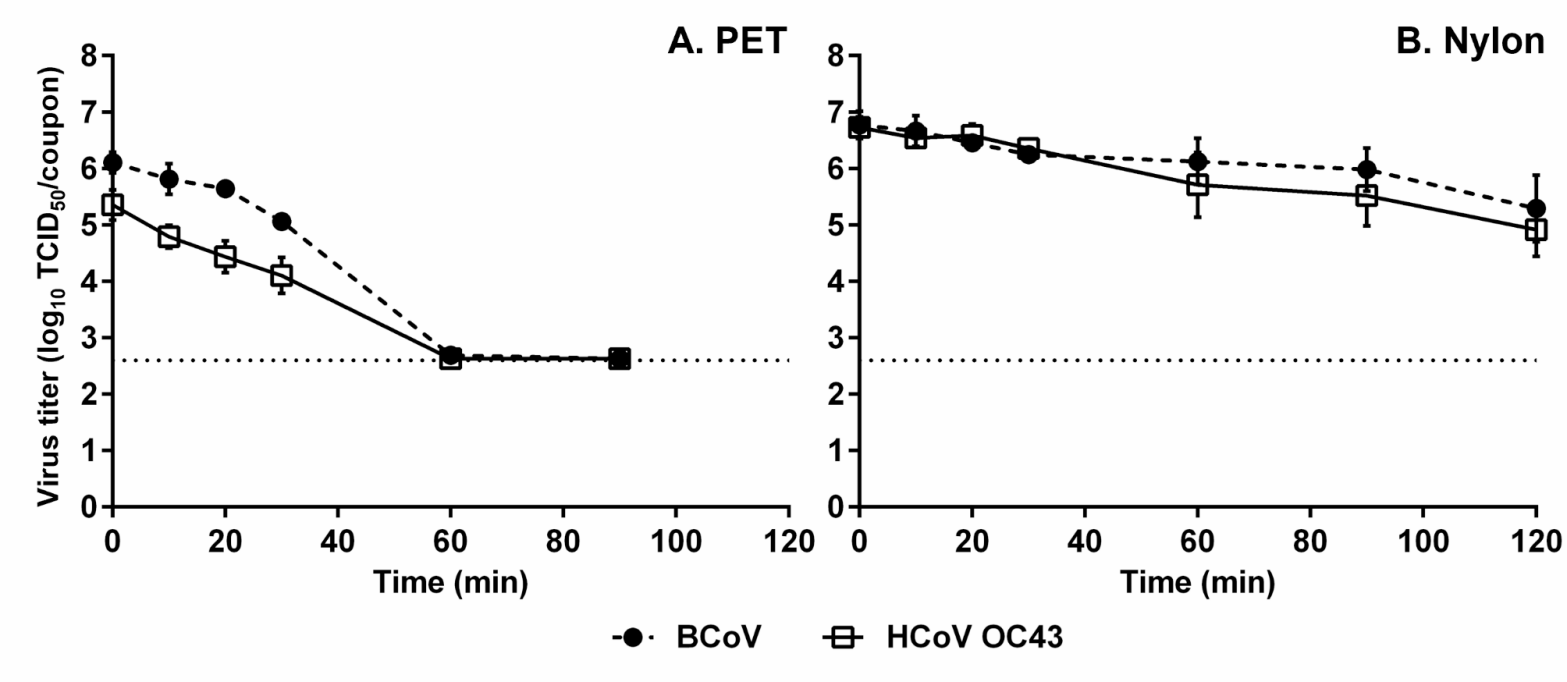
Two infectious coronaviruses on PET (A) and nylon (B) carpets. Data are expressed as mean ± standard deviation (SD) from six replicates in two trials. Dotted lines indicate the detection limit at 2.6 log_10_ TCID_50_

After 0, 10, 20, 30, 60, and 90 min of inoculation, the titers of BCoV on PET carpet were 6.1, 5.8, 5.6, 5.1, 2.7, and < 2.6 log_10_ TCID_50_/coupon, respectively, and the titers of HCoV OC43 were 5.4, 4.8, 4.4, 4.1, < 2.6, and < 2.6 log_10_ TCID_50_/coupon, respectively (Fig. 1A). In contrast, 6.8, 6.7, 6.5, 6.2, 6.1, 6.0 and 5.3 log_10_ TCID_50_/coupon of BCoV were detected on nylon carpet after 0, 10, 20, 30, 60, 90, and 120 min, respectively (Fig. 1B), and 6.7, 6.5, 6.6, 6.4, 5.7, 5.5 and 4.9 log_10_ TCID_50_/coupon of HCoV OC43, respectively.

### Persistence of coronavirus whole genome on carpet

**Fig. 2:**
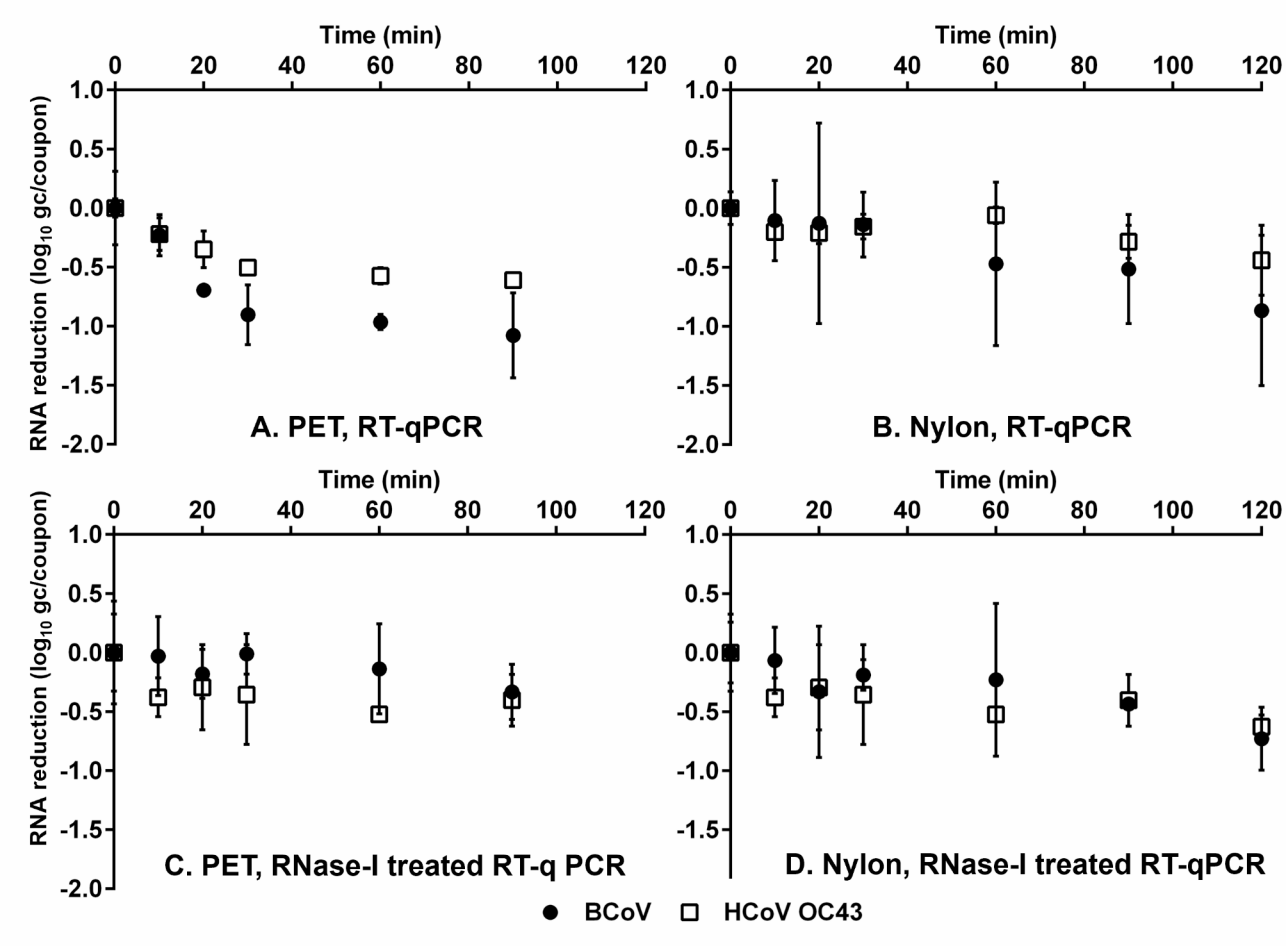
RNA copy reduction of BCoV and HCoV OC43 on PET (A) and nylon (B) carpets, and RNase I-treated BCoV and HCoV OC43 on PET (C) and nylon (D) carpets. Data are expressed as mean ± standard deviation (SD) from six replicates in two trials

The immediate recovery rate of BCoV gRNA from PET and nylon carpet was 52.32% and 57.64%, respectively, as compared with 40.84% and 28.54% for HCoV OC43 gRNA from PET and nylon carpet, respectively (Table 1). On PET carpet, gRNA of BCoV decreased by 0.23, 0.69, 0.90. 0.97, and 1.08 log_10_ genome copies (gc)/coupon, respectively, and a 0.22, 0.35, 0.50, 0.57, and 0.61 log_10_ gc/coupon reduction of HCoV OC43 after 10, 20, 30, 60, and 90 min, respectively (Fig. 2A). On nylon carpet, gRNAs of BCoV showed a 0.11, 0.13, 0.14, 0.47, 0.52, and 0.87 log_10_ gc/coupon reduction after 10, 20, 30, 60, 90, and 120 min, respectively, while gRNA of HCoV OC43 had a 0.20, 0.21, 0.16, 0.06, 0.28, and 0.44 log_10_ gc/coupon reduction, respectively (Fig. 2B). Based on first-order decay kinetics, the *k* values of BCoV and HCoV OC43 whole gRNA on PET carpet were 1.19 and 0.67 h^− 1^, respectively, and on nylon carpet were 0.86 and 0.27 h^− 1^, respectively (Table 2).

**Table 2:** First-order decay rate constants *k* for whole gRNAs and unexposed gRNAs of BCoV and HCoV OC43 on PET and nylon carpet

Virus	Decay rate constants k (h^− 1^) ^a^			
	Whole gRNA		Unexposed gRNA	
	PET carpet	Nylon carpet	PET carpet	Nylon carpet
BCoV	1.19 ± 0.62^A/A^	0.86 ± 0.29^A/A^	0.61 ± 0.20^A/A^	0.84 ± 0.21^A/A^
HCoV OC43	0.67 ± 0.30^A/A^	0.27 ± 0.14^A/A^	0.28 ± 0.33^A/A^	0.43 ± 0.20^B/A^

^a^ Data are expressed as mean ± standard deviation (SD), calculated based on triplicates at each of the five sampling time intervals. Values with different letters within/across carpet type (e.g., A/A, B/A) for the whole gRNA or unexposed gRNA indicate significant difference (*p* < 0.05) in Tukey’s test grouping

### Persistence of coronavirus unexposed genome on carpet

Compared to the whole gRNA, unexposed gRNA wrapped inside viral capsids, which representing the intact capsid, was decreased more slowly on PET carpet by a 0.03, 0.18, 0.10, 0.14, and 0.33 log_10_ gc/coupon reduction of BCoV, and a 0.38, 0.29, 0.36, 0.52, 0.40 log_10_ gc/coupon reduction of HCoV OC43 after 10, 20, 30, 60, and 90 min, respectively (Fig. 2C). On nylon carpet, gRNA of BCoV was decreased by 0.07, 0.33, 0.19, 0.23, 0.43, and 0.73 log_10_ gc/coupon reduction after 10, 20, 30, 60, 90, and 120 min, respectively, whereas gRNA of HCoV OC43 had a 0.38, 0.29, 0.36, 0.52, 0.40, and 0.63 log_10_ gc/coupon reduction, respectively (Fig. 2D). As the exposed gRNA was removed by RNase I, the *k* values of unexposed gRNA from BCoV and HCoV OC43 were 0.61 and 0.28 h^− 1^ on PET carpet, respectively, and 0.84 and 0.43 h^− 1^on nylon carpet, respectively (Table 2).

### Efficacy of steam vapor against coronaviruses on nylon carpet

**Fig. 3:**
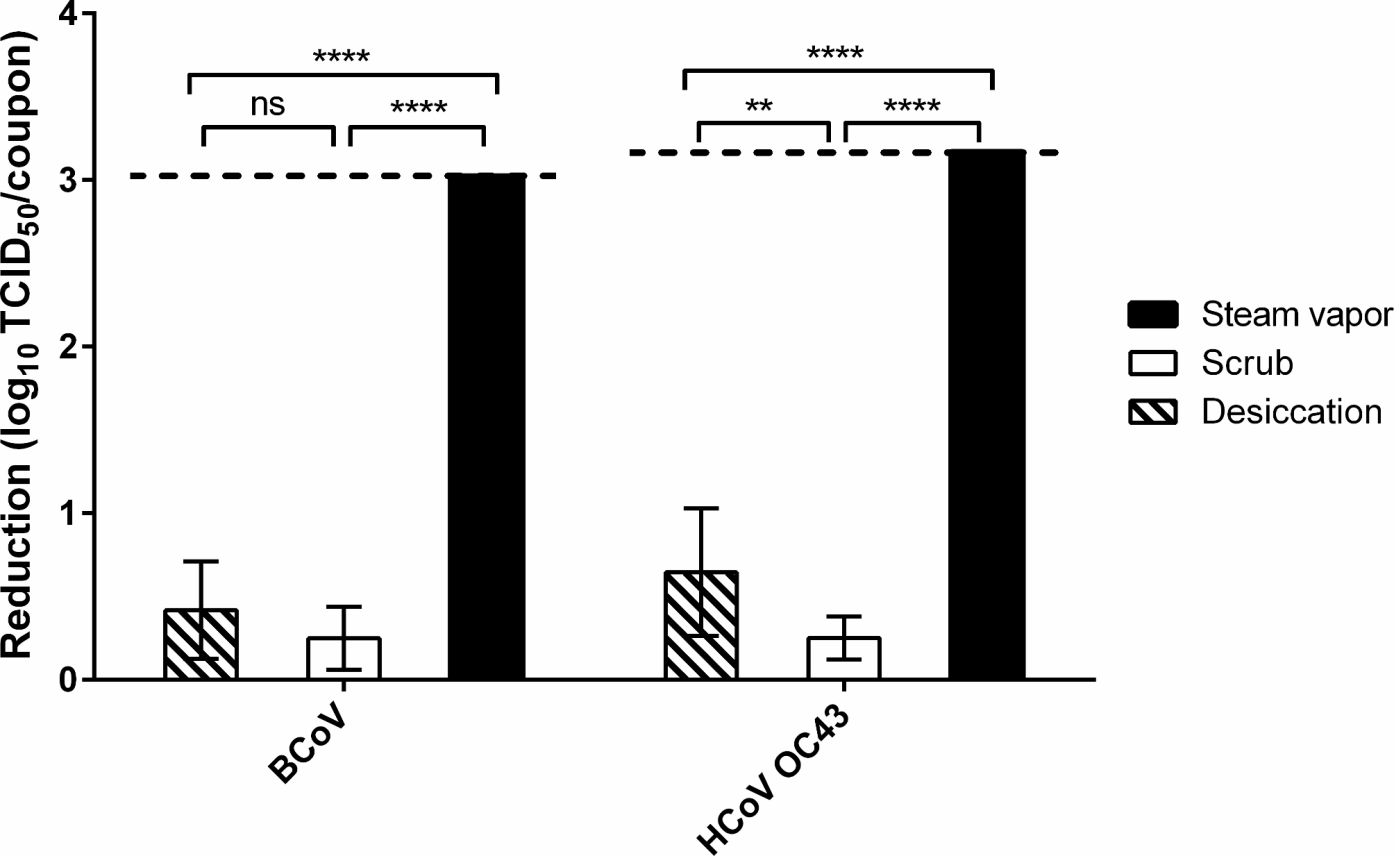
Efficacy of steam vapor against two coronaviruses on nylon carpet. Data are expressed as mean ± standard deviation (SD) from ten replicates in two trials. The dash lines indicate the detection limit for titer reduction (BCoV >3.0, HCoV OC43 >3.2 log_10_ TCID_50_/coupon). The *p*-value among treatments for each virus was ≥ 0.05 (ns), < 0.01 (**) and < 0.0001 (****)

Following a 1-hour drying period on nylon carpet, desiccation resulted in a 0.4 log_10_ TCID_50_ reduction for BCoV and 0.6 log_10_ TCID_50_/coupon reduction for HCoV OC43 (Fig. 3). The temperature of steam vapor from the steam cleaner head reached 99.44 ± 0.98 °C. Subsequent treatment with steam vapor inactivated both BCoV and HCoV OC43 across all carpet coupons in 15 s, indicating virucidal efficacy of this approach. Specifically, steam vapor achieved a > 3.0 log_10_/coupon TCID_50_ reduction of BCoV and > 3.2 log_10_ TCID_50_/coupon of HCoV OC43 on nylon carpet. Mechanical scrubbing alone resulted in a 0.2 log_10_ TCID_50_/coupon reduction for both BCoV and HCoV OC43.

### Carpet absorption capacity

PET fiber (0.1 g) absorbed up to 0.65–0.70 mL of safranin solution (Table 3). In contrast, 0.1 g of nylon fibers reached saturation, retaining only 0.50–0.55 mL of safranin solution. When 0.55 mL of safranin solution was added, nylon fibers had a greater amount of residual liquid than did PET fibers. Therefore, PET fibers tested exhibited a higher degree of hydrophilicity compared to nylon fibers.

**Table 3:** Absorptive capacity of carpet fibers

Sample^a^	Vol added (mL)	Residual wt (µg)^b^
PET	0.500	6.2 ± 1.3^A^
	0.550	7.2 ± 1.8^AB^
	0.600	8.5 ± 2.3^AB^
	0.650	12.5 ± 5.2^BC^
	0.700	15.3 ± 5.2^C^
Nylon	0.400	5.7 ± 1.0^A^
	0.450	8.8 ± 1.8^A^
	0.500	9.7 ± 2.5^AB^
	0.550	16.3 ± 6.0^B^
	0.600	23.5 ± 6.9^C^

^a^ Carpet fiber samples were 0.1 g each

^b^ Data are expressed as mean ± standard deviation (SD) from triplicates at each of 2 independent experiments. Values of residual weight with different letters for the same fiber (e.g., A, B) indicate significant difference (*p* < 0.05) in Tukey’s test grouping

## Discussion

The persistence of two betacoronaviruses on PET and nylon carpets and the efficacy of steam disinfection of both coronaviruses on nylon carpet were investigated. In our study, more viable BCoV was recovered from both carpet types than was HCoV OC43, while BCoV was more resistant to desiccation on surfaces with a slower loss in infectivity. The more hydrophilic PET carpet caused significant loss of infectivity for both coronaviruses and possible viral capsid damage, highlighting that infection assays are more accurate in assessing coronavirus infectivity loss than RT-qPCR assays. Our persistence results suggest risk of viral transmission may be low after the contamination of PET carpet by coronaviruses due to the rapid loss of infectivity. Conversely, both viruses declined slowly on nylon carpet. Lastly, steam vapor was efficacious enough to eliminate both coronaviruses within 15 s, indicating the potential of steam vapor as a rapid and effective disinfectant against coronaviruses including SARS-CoV-2 on porous surfaces like nylon carpet.

Coronaviruses, such as SARS-CoV-2, are hard to recover from environmental surfaces, with the recovery rate affected by the surface material and recovery methods [25]. For example, Riddell and colleagues [10] recovered viable coronaviruses by repeated pipetting with an approximate 3-log loss from cotton cloth. In addition, other studies also revealed the adverse effect of recovery media, composition of surfactants and elution methods on the recovery rate of coronaviruses [32]. To our knowledge, no study has reported the recovery of coronaviruses from carpet materials. This is possibly attributed to the fact that surfactants and mechanical agitation used for recovery could chemically and physically affect the phospholipid layer and spike proteins on viral envelopes [25]. The envelope structure of coronaviruses plays a critical role in its attaching and entering host cells, hence, any damage to the envelope structure including the phospholipid layer and spike proteins might lead to loss of infectivity [25, 33]. In our study, BCoV and HCoV OC43 were successfully recovered, with less than 1 log_10_ TCID_50_ reduction from PET and nylon carpet using the method reported previously for norovirus [22]. However, more HCoV OC43 was lost in recovery than BCoV, suggesting BCoV was more resistant to the recovery method. Additionally, the higher gRNA recovery rates immediately following inoculation suggest the effectiveness of our elution method (Table 1).

HCoV OC43 was less persistent on PET fabrics with > 3 log_10_ TCID_50_ reduction after 1 h at 35 °C [34]. A similar result was observed in our study as viable BCoV and HCoV OC43 were rapidly inactivated on PET carpet, reaching the detection limit within one hour. In comparison, both coronaviruses survived longer on nylon carpet with ≤ 2 log_10_ TCID_50_ reduction of viable viruses after two hours. Different levels of persistence of these two coronaviruses were also observed on plastic and vinyl surfaces [26]. In contrast, SARS-CoV-2 has been shown to survive longer for 1–7 days at 20–30 °C on cotton cloth, and HCoV OC43 survived for 2 days at room temperature [10, 35]. The correlation between persistence of coronaviruses and surface material and construction could be explained by the fact that coronaviruses were rapidly inactivated on carpet fabric with a faster water absorption [35], which our absorption data also supported (Table 3). Viruses can easily cover the surface of hydrophilic fibers, resulting in larger surface area of exposure to desiccation, whereas the high surface hydrophobicity promotes virus aggregation and concentration to protect viruses within the aggregates [12, 36]. Additionally, the rate of decline for unexposed gRNAs was significantly slower than that of whole gRNAs for both BCoV and HCoV OC43 on PET carpet. This difference was not observed on nylon carpet. Therefore, coronavirus capsids are more likely to be damaged on PET carpet than on nylon carpet, which is more hydrophobic.

Infectivity assays are not always used in studies regarding non-porous and porous environmental surfaces, partially due to the challenges associated with the recovery of viable viruses from surfaces [25, 37, 38]. In our study, the reduction of coronavirus gRNA on PET carpet occurred at a slower rate (< 1 log_10_ gc h^− 1^) than did the decline in viral infectivity (> 2.8 log_10_ TCID_50_ h^− 1^) at room temperature. Coronavirus gRNAs are encased within viral capsids, protecting the structure [39]. Environmental factors can facilitate the disruption of viral envelopes and capsids before acting upon the gRNAs. As such, relying solely on gRNA detection may not accurately reflect the persistence of coronavirus and its disinfection efficacy.

Interestingly, we found that unlike infectivity, whole gRNAs of HCoV OC43 degraded slower than BCoV on both PET and nylon carpet (Fig. 2A-B). This is likely due to the capsid protein of HCoV OC43 being more resistant to desiccation. As most coronaviruses only share 43% identity on the structural protein-coding region [40], it is not surprising that the HCoV OC43 capsid is more resistant than BCoV to desiccation but still within the same order of magnitude. Apart from structural proteins, HCoV OC43 shared similar spike proteins with BCoV, particularly both viruses having a deletion within the S1 subunit of the spike protein [41]. While oxidation-sensitive amino acids (i.e., tyrosine, tryptophan, and histidine) are abundant in the receptor binding domain of the spike proteins [42], the spike proteins of HCoV OC43 are more sensitive than BCoV to oxidation [42], resulting in the significant loss of infectivity for HCoV OC43 when drying on PET carpet.

Heat is an important factor for the persistence and disinfection efficacy of viruses. More than 3 log_10_ TCID_50_ of MERS-CoV, SARS-CoV, and SARS-CoV-2 were reduced in cell culture medium when exposure to temperatures ≥ 60 °C was as short as 15 min [43–45]. However, such heat inactivation of coronaviruses has been investigated in suspension only. Because BCoV and HCoV OC43 were reduced below the detection limit during a 1-h drying on PET carpet, we investigated the efficacy of steam vapor against both coronaviruses only on nylon carpet. Steam vapor was efficacious against both coronaviruses on nylon carpet, achieving > 3 log_10_ TCID_50_/coupon reduction within 15 s (Fig. 3). This robust virucidal activity can be attributed to the potential of steam vapor reaching temperatures as high as 99.44 ± 0.98 °C on carpet, while mechanical forces, like scrubbing without heat could only inactivate a few coronavirus particles. Moreover, steam vapor has been proven to be safer for use on nylon carpets, with minimal impact on carpet properties [46]. However, it’s important to acknowledge that the efficacy of disinfectants including steam vapor could be influenced by other factors [47]. Specifically, fiber construction, including characteristics like looped or pile cut, materials employed, and fiber length, all could affect the performance of steam vapor [22]. Due to the scope of this study, we were unable to comprehensively evaluate these factors or perform a complete kill analysis during steam treatment. This limitation leaves room for further investigation in future research.

## Conclusion

In summary, this study examined the persistence of two betacoronaviruses, BCoV and HCoV OC43, on PET and nylon carpet. Our results showed that more viable BCoV was recovered from both carpets than was HCoV OC43. Additionally, viable viruses were rapidly inactivated on PET carpet, but titers remained relatively stable on nylon carpet. Furthermore, we confirmed that steam vapor is an effective disinfectant against both coronaviruses on nylon carpet. This study addressed the critical issue of disinfecting carpet contaminated with coronavirus. These results can be used to inform effective disinfection of human and animal coronavirus on porous materials. Additionally, in the absence of biosafety level-3 facilities, both BCoV and HCoV OC43 can be used to screen disinfectants for efficacy against SARS-CoV-2.

## Data Availability

No datasets were generated or analysed during the current study.

## Abbreviations

BCoV: Bovine coronavirus
HCoV OC43: Human coronavirus OC43
MERS-CoV: Middle East Respiratory Syndrome coronavirus
SARS-CoV: Severe acute respiratory syndrome coronavirus
CDC: United States Centers for Disease Control and Prevention
PET: Polyethylene terephthalate
DMEM: Dulbecco’s Modified Eagle Medium
TCID_50_: Median tissue culture infectious dose
PBS: Phosphate buffer saline
gRNA: Genome RNA
RT-qPCR: Reverse transcription quantitative polymerase chain reaction
